# Factors influencing participation of elderly population in mass drug administration for lymphatic filariasis: a cross-sectional study

**DOI:** 10.3389/fphar.2024.1429653

**Published:** 2024-11-06

**Authors:** Muhammed Jabir, Vijayakumar Balakrishnan, Kaliannagounder Krishnamoorthy, Ashwani Kumar, Philip Raj Abraham

**Affiliations:** ^1^ Division of Epidemiology and Operational Research, ICMR-Vector Control Research Centre, Puducherry, India; ^2^ Division of Biostatistics and VBD Modelling, ICMR-Vector Control Research Centre, Puducherry, India; ^3^ ICMR-Vector Control Research Centre, Puducherry, India; ^4^ Saveetha Institute of Medical and Technical Sciences, Saveetha University, Thandalam, Tamil Nadu, India; ^5^ Unit of Molecular Epidemiology, ICMR-Vector Control Research Centre, Puducherry, India

**Keywords:** lymphatic filariasis, mass drug administration, elderly population, participation, compliance, coverage

## Abstract

**Background:**

The success of mass drug administration (MDA) for lymphatic filariasis (LF) elimination relies on achieving a participation rate of at least 65% within the endemic community. However, participation of sub-population in the community varies and a significant treatment gap among the elderly population, remains to be addressed. The present study explores the factors influencing the elderly participation in MDA and propose possible solutions to bridge the gap.

**Methods:**

A cross-sectional study of individuals aged 60 years and above was conducted from August to December 2023 in Yadgiri district of Karnataka, which is endemic for LF. The participants were interviewed using a structured questionnaire, focusing on the perception about LF and MDA and drug consumption behaviours. STATA 14 software was used to analyse the data. We used a logistic regression model to determine the factors influencing drug consumption.

**Results:**

The study included 315 elderly individuals with a mean age (SD) of 67.4 (6.2) years. Although, 58.4% of them received the drugs during the last round of MDA in 2023, only 40.6% consumed it. The drug refusal rate was 19.4%. Fear of side effects (22.9%) was cited as the primary reason for not accepting the drugs. Weak perception of LF transmission risk (25.7%) and mistrust of drug safety (42.5%) were reported as reasons for non-compliance. Logistic regression identified significant associations, including residence (peri-urban: OR = 6.80), chronic disease (diabetes: OR = 2.89), trust on drug safety (OR = 16.27), and opinion of neighbours (OR = 5.35).

**Conclusion:**

Participation of elderly population in MDA was suboptimal (40.6%). Tailored interventions to improve consumption such as addressing misconceptions, building trust in MDA and effective monitoring and management of adverse events are vital to enhance their participation. The National Programme should have specific guidelines and strategies to address this issue to improve their participation in MDA for elimination of LF.

## Introduction

Lymphatic filariasis (LF) remains a significant public health concern in India, with the country accounting for 61.5% of the 657 million global population requiring MDA for the elimination of LF (World Health Organization, 2024). This disease, caused by filarial nematodes and transmitted through infected mosquito bites, imposes a significant healthcare burden on the country (World Health Organisation, 2023). The physical morbidity resulting from lymphedema and hydrocele, coupled with the social stigma and psychological distress experienced by those affected, could perpetuate a vicious cycle of poverty and suffering (Sabesan et al., 2022; Zeldenryk et al., 2011; Perera et al., 2007).

India launched the National Programme to Eliminate LF in 2004, targeting 202 known endemic districts in the country. The nation’s strategy for disease elimination involves preventive chemotherapy through annual MDA in the endemic districts and morbidity management and disability prevention (MMDP), aligning with WHO’s recommendations (National Centre for Vector Borne Diseases Control). As of 2023, 176 districts were under MDA, with the country aiming to achieve LF elimination by 2030 (National Centre for Vector Borne Diseases Control, 2023).

The WHO endorsed the use of a triple-drug regimen (IDA: Ivermectin, Diethylcarbamazine and Albendazole) as an alternative MDA regimen to eliminate LF (World Health Organization, 2017). This strategy aims at effective coverage of at least 65% of the total population and require only fewer rounds of MDA (World Health Organization, 2019). WHO has provided a provisional IDA impact survey based on microfilariae (Mf) prevalence as the epidemiological indicator. As many as 63 districts with persistent LF transmission in India were under IDA in 2023 and a few districts continued MDA beyond 2 recommended rounds due to sub-optimal impact. Suboptimal coverage is a major challenge leading to the persistence of Mf carriers. Key factors contributing to suboptimal coverage include migration, hard-to-reach communities, urban poor, and other vulnerable groups (Roy et al., 2013; Babu and Babu, 2014; Banerjee et al., 2019). Modelling studies have indicated how low coverage significantly reduces the likelihood of achieving elimination targets by 2030 (Dyson et al., 2017). Furthermore, the non-compliers of MDA remain untreated and if infected, could serve as a reservoir of infection and contribute to transmission. Under these circumstances, achieving the elimination target is a programmatic challenge (Coutts et al., 2017; Willis et al., 2020).

The present study focuses on the participation of the elderly population (individuals aged >60 years) (Ministry of Social Justice and Empowerment, 2011) in MDA for LF. The elderly in rural India face unique challenges, including high illiteracy rates, poverty, social discrimination, and low awareness of social schemes and services (United Nation’s Population Fund 2011; Chandramouli, 2011; Ministry of Statistics and Programme Implementation, 2016; Ministry of Statistics and Programme, 2015). Studies shows that the participation of the elderly in healthcare activities is shaped by a variety of social, economic, cultural and political factors (Srivastava and Gill, 2020; Sahoo et al., 2021). Key determinants of their healthcare-seeking behaviour include autonomy in treatment decisions, disease perception, education level, economic status, and access to and awareness of available healthcare services (Braimah et al., 2023; Banerjee, 2021). Negative healthcare-seeking behaviour is prevalent among the elderly, driven by the perception of having spent most of their life and nearing the end (Arjyal et al., 2023). In the context of LF-MDA, coverage evaluation studies conducted internationally report an association between older age (≥60 years) and non-consumption of drugs (Dickson et al., 2021). While Indian studies often lack specific coverage data for the elderly, some indicate significantly lower participation rates in this age group. A recent study in two districts of Jharkhand found a non-consumption rate of 45.9% among individuals aged 60 and older (Kumar et al., 2023).

Several articles on the independent Coverage Evaluation Survey (CES) analysed the coverage and the reasons for non-participation in MDA in India. The present study is probably the first of its kind on the compliance of the elderly population for LF-MDA and the results can be used to develop appropriate strategies to bridge the coverage gap.

## Methods

### Study setting

**FIGURE 1:**
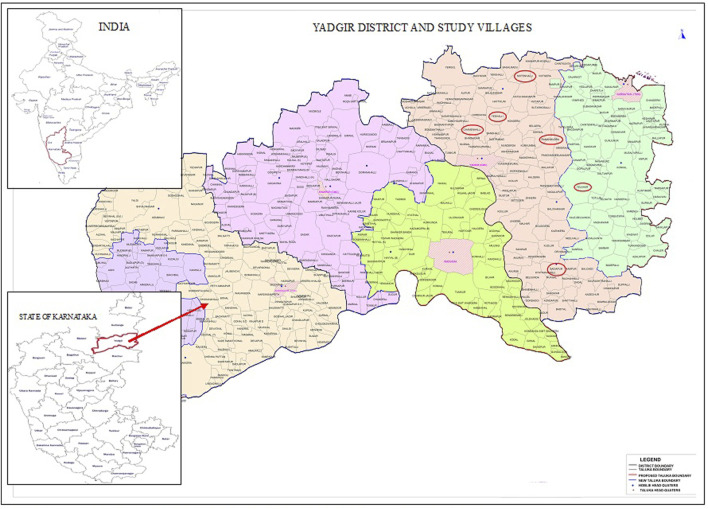
Map showing the study villages (red color) in Yadgir district.

This study was conducted in six randomly selected villages from an LF endemic block of Yadgir district, Karnataka (Figure 1). The district has a population of 1.2 million, with 62.8% engaging in agriculture and allied activities (Chandramouli, 2011). Being an economically challenged area, seasonal migration is prevalent. The district is endemic for bancroftian filariasis transmitted by *Culex quinquefasciatus* mosquitoes (Kuttiatt et al., 2020). MDA for LF elimination in the district was started in 2004 and completed 15 rounds with DA (diethylcarbamazine (DEC)+Albendazole). In 2019, an MDA with an IDA drug regimen was implemented and so far, three annual rounds have been completed (National Centre for Vector Borne Diseases Control). Independent assessment of drug coverage, conducted in 2015 and 2018 showed a consumption rate of 56.2% and 75.4% (Havale, 2015; Shetty et al., 2012) respectively.

### Study design

This community-based cross-sectional study was conducted between August and December 2023. Six villages; namely Ramasamudra, Yelheri, Saidapur, Motnahalli, Chamanahalli and Yedahalli were selected from an endemic health block (Figure 1). A sample size of 267 was initially calculated with an assumed proportion of drug compliance (drug consumed) of 50% among the elderly population, an absolute precision error of 6% and a confidence interval of 95%. The final sample size was arrived at 315 by assuming a non-response rate of 15%. A list of elderly population with age was provided by the community drug administrators (CDA) and the participants were randomly selected from the list. The inclusion criteria included 1) individuals aged ≥60 years 2) permanent residents of the study site and 3) willing to provide informed consent for participation. Exclusion criteria were 1) individuals below the age of 60 2) those suffering from cognitive impairments and 3) severely ill.

### Tool and data collection

Participants were interviewed using a pre-tested semi-structured questionnaire comprising five major sections. The first two sections were on demographic characteristics and self-reported disease of the study participants. The third and fourth sections were on knowledge and perception of LF and MDA. The last section was on participation in MDA and associated determinants. Interviews were conducted by a sociologist, in the local language (Kannada) with interpretation by trained field workers. Personal interviews were conducted with all the eligible participants available at the time of visit by the research team. The interview lasted for 30–40 min per individual. The details on drug consumption were verified from the family registers of CDAs. Informal discussion was also held with community drug distributors and observations were summarised.

### Data analysis

Data were fed in an Excel spreadsheet and all statistical analyses were done using STATA 14.2 (Texas, United States). All categorical variables were presented as frequencies with percentages in tables and figures. Univariate and multiple logistic regression analyses were used to find out factors associated with non-compliance with MDA. In the adjusted model, variables from the unadjusted model with a p-value of less than 0.25 were included. The goodness-of-fit of the model was assessed using the Hosmer-Lem show test. A p-value less than 0.05 was considered statistically significant for the final adjusted logistic regression model. The knowledge scores of the participants were computed based on binary responses (“yes” or “no”) regarding awareness of LF transmission, symptoms and preventive measures. A score of “one” was assigned for each affirmative response and “zero” otherwise, with the maximum attainable score being 4. Non-compliance rate was defined as the percentage of individuals who self-reported not swallowing the drugs provided during the MDA campaigns.

## Results

### Demographic profile

This study involved 315 elderly individuals with a higher proportion of females (66.7%) (Table 1). The mean (+SD) age of the respondents was 67.44 (±6.23) years and most of them were in the age class of 60–70 years (80.3%). The majority of the respondents belonged to Hindu religion (82.2%), while others belonged to Christian (9.8%), Muslim (7%) and Jains (1%). While 85.1% of the respondents did not have any formal education, 10.5% of them completed primary or upper primary school, and 3.2% had secondary education and higher. The majority (82.8%) reside as joint families (living with sons/daughters) while a smaller proportion live in nuclear families. None of the participant declined to participate in the study as ASHA workers in the respective villages assisted the survey.

**TABLE 1:** Demographic characteristics.

Variables	Category (N = 315)	n (%)
Gender	Male	105 (33.3)
	Female	210 (66.7)
Age (in completed years)	60–70 years	253 (80.3)
	71–80 years	52 (16.5)
	81 and above	10 (3.2)
	Mean (±SD)	67.44 years (±6.23)
Religion	Hindu	259 (82.2)
	Christian	31 (9.8)
	Muslim	22 (7)
	Jain	3 (1)
Area of residence	Rural	240 (76.2)
	Peri-urban	75 (23.8)
Education	No formal education	268 (85.1)
	Primary	26 (8.2)
	Middle	7 (2.2)
	High school	10 (3.2)
	Graduation	4 (1.3)
Type of family	Joint family	261 (82.9)
	Nuclear family	54 (17.1)

### Self-reported diseases

A total of 127 (40.3%) respondents reported having one or more chronic diseases. Hypertension was reported by 28.6% of participants, followed by diabetes (16.8%). Other reported diseases included cardiovascular problems (3.5%), asthma (1.9%) and cancer (0.6%). Signs of LF were found in 15 (4.8%) respondents.

### Knowledge and perceptions on LF and MDA

While almost everybody reported having heard about LF, only 66.7% possessed correct knowledge on the involvement of mosquitoes in the transmission. When asked about the signs of LF, 82% of the respondents were able to identify at least one correct sign (swelling of the legs and hydrocele as signs of LF). Only about half of the respondents (46%) were aware of preventive measures against LF (Table 2). The literate individuals had a higher knowledge of LF and MDA (78.7%) compared to individuals with no formal education (55.2%) and the difference was statistically significant (*p* = 0.003). Similarly, among religious groups, non-Hindus (including Christians, Jains, and Muslims) had more knowledge on LF and MDA (76.8%) compared to Hindus (55%) and this difference is significant (*p* = 0.002). However, the level of knowledge on LF does not differ statistically (*p* = 0.746) between males (60%) and females (58.1%).

**TABLE 2:** Knowledge and perception of LF and MDA.

Variables	Category	n (%)
Knowledge		
Heard about LF	Yes	292 (92.7)
	No	23 (7.3)
Correct knowledge on the involvement of mosquitoes in the LF transmission	Yes	210 (66.7)
	No	105 (33.3)
Correct knowledge on the signs of LF disease (Swelling of limbs, hydroceles)	Yes	258 (82)
	No	57 (18)
Correct knowledge on the preventive measures (MDA, mosquito control, mosquito nets) against LF	Yes	145 (46)
	No	170 (54)
Perception		
Is LF a serious disease?	Yes	254 (80.6)
	No	4 (1.3)
	Unsure	57 (18.1)
Are you at risk of LF?	Yes	81 (25.7)
	No	115 (36.5)
	Unsure	119 (37.8)
Whether MDA drugs are safe?	Yes	134 (42.5)
	No	22 (7)
	Unsure	159 (50.5)
Will you take MDA drug in future MDA rounds?	Yes	149 (47.3)
	No	0
	Unsure	166 (52.7)

Most of the respondents (80.6%) perceived LF as a disease of concern, whereas only a few (25.7%) could recognize that they are at risk of LF infection. Only 42.5% of the respondents expressed that the drugs were safe. Responding to their willingness to take drugs in future MDA campaigns, only 47.3% were affirmative. Although literate individuals expressed a more positive perception (44.7%) towards LF and MDA compared to individuals with no formal education (33.2%), this difference was not statistically significant (*p* = 0.128). Similarly, the perception was not differing significantly between males and females (*p* = 0.559) as well as between religious groups (*p* = 0.891).

### Drug consumption in MDA among the elderly population

Most of the respondents (90.8%) reported have been offered MDA drugs at least once in their lifetime. Regarding the frequency of MDA receipt, 81.6% reported receiving MDA drugs at least three times, and a small proportion of them reported receiving twice (6.7%) and once only (2.53%). Among those who received the drugs (n = 286) in previous rounds, the majority (83.5%) reported having consumed the drug, with a significant proportion (27.4%) reporting adverse effects (Table 3).

**TABLE 3:** Consumption status in the previous and last round of MDA.

Variables	Category	n (%)
Drug consumption in previous rounds out of total 18 rounds of MDA		
Ever received the MDA drug	Yes	286 (90.8)
	No	29 (9.2)
No. of times MDA drug received	One time only	8 (2.53)
	Two times only	21 (6.7)
	Three or more than 3 times	257 (81.6)
Ever consumed MDA drug (n = 286)	Yes	263 (83.5)
	No	52 (16.5)
Ever experienced side effects (n = 263)	Yes	72 (27.4)
	No	191 (72.6)
Drug consumption in the last round of MDA (2023)		
Received the drug in the last round of MDA	Yes	184 (58.4)
	No	131 (41.6)
Consumed the drugs in the last round of MDA	Yes	128 (40.6)
	No	187 (59.4)
Reason for consumption (N = 128)	To protect myself from disease	101 (78.9)
	Instructed by drug administrator	8 (6.3)
	Instructed by family and others	19 (14.8)

**FIGURE 2:**
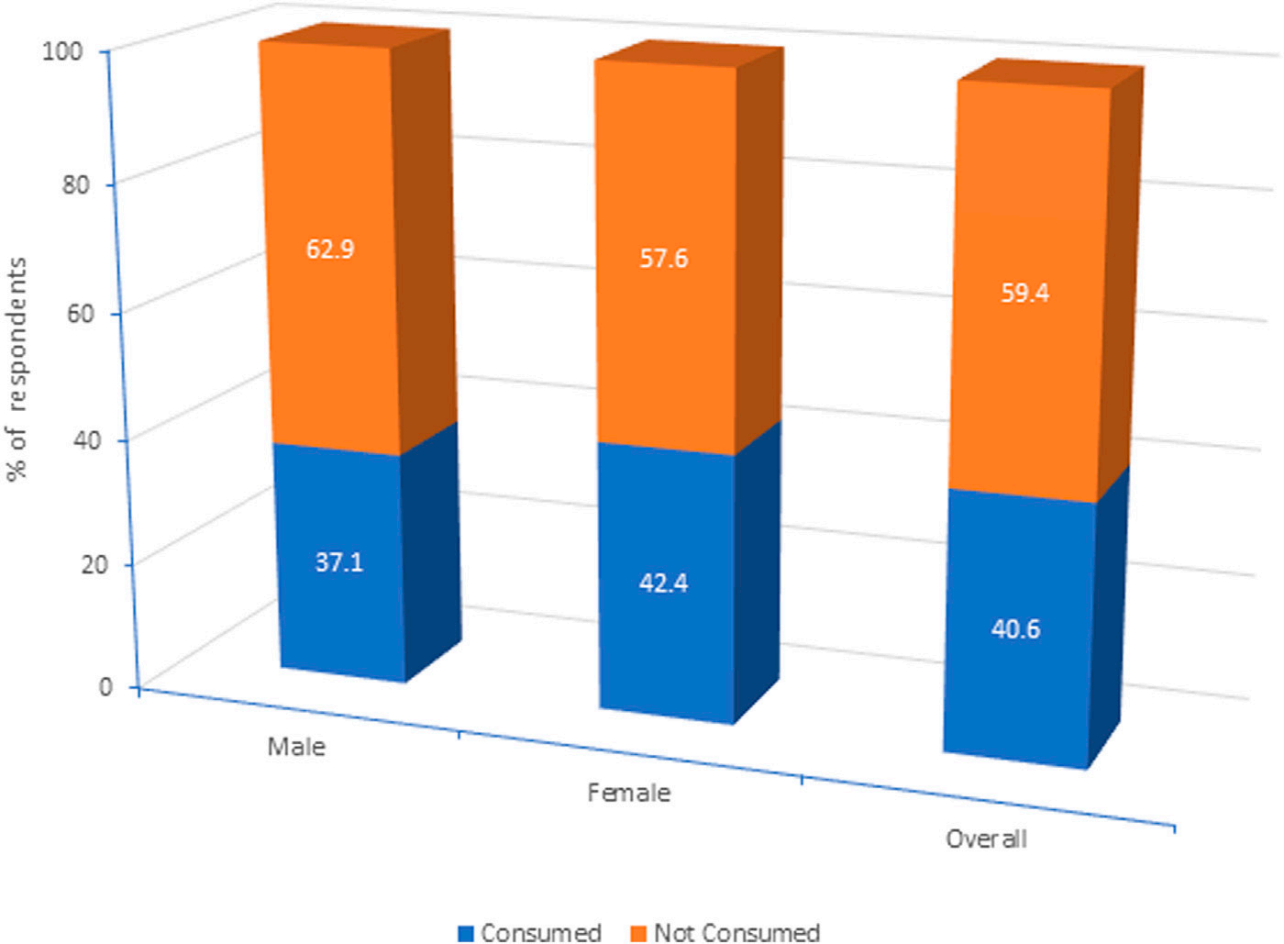
Gender-wise consumption of MDA drugs.

In the most recent round of MDA in 2023, the coverage of the elderly population was only 58.4% and consumption was 40.6%. Reasons for consumption by those who received included protection from the disease (78.9%), instructions from drug administrators (6.3%), and advice from family or others (14.8%) (Table 3). The consumption of the drugs among females was slightly higher (45.4%) compared to males (37.1%). However, the gender difference was not statistically significant (*p* = 0.373) (Figure 2).

### Reasons for non-receipt and consumption of drugs in the last round of MDA

**FIGURE 3:**
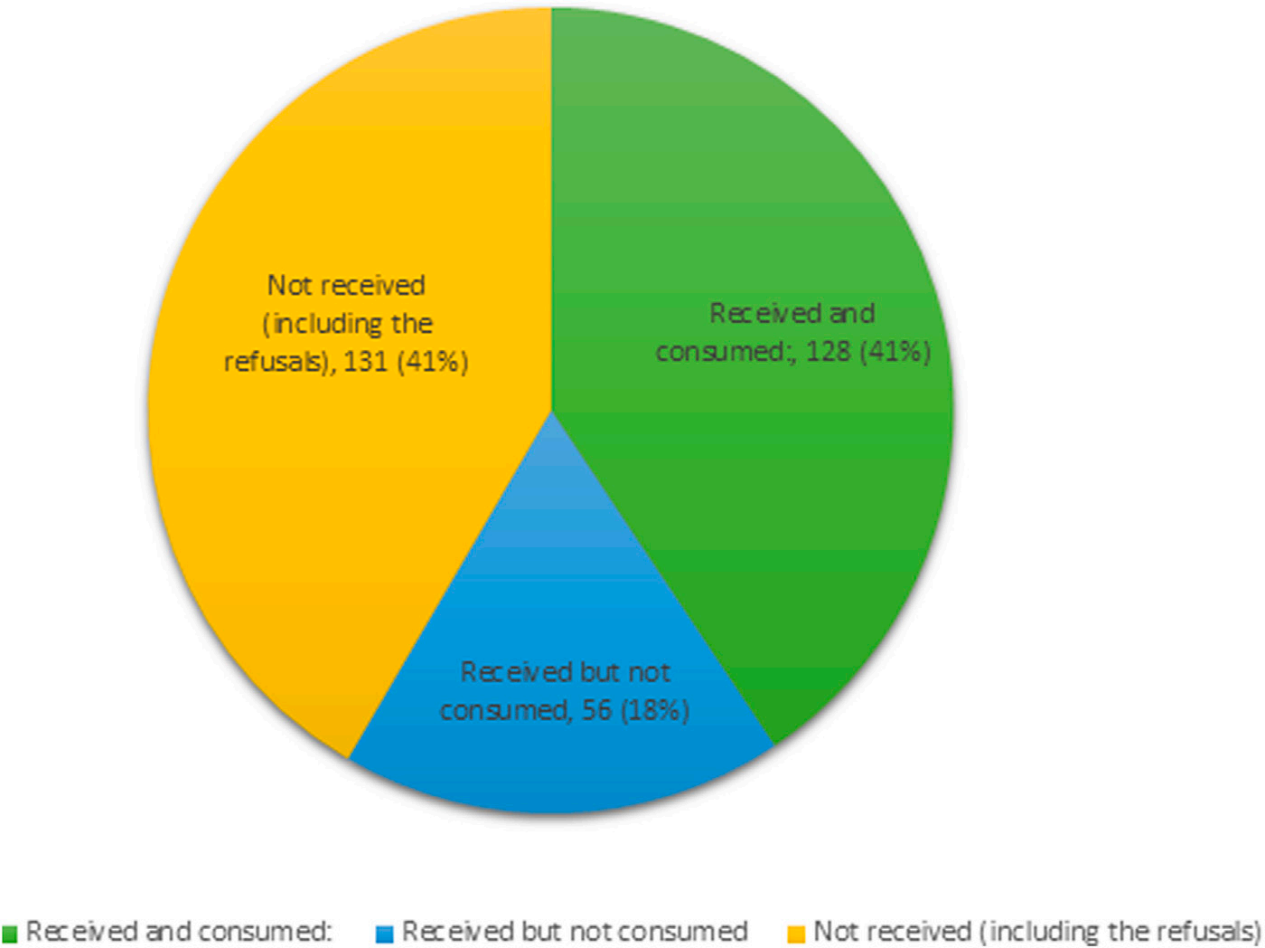
Drug consumption status.

Out of 315 respondents, 184 (58.4%) reported having received the drugs. Of those who received the drugs, 128 (69.6%) consumed them (Table 3). There was a coverage gap of 41% and a consumption gap of 30.4% (Figure 3).

Among the 187 individuals who did not consume the drugs, 70 (37.4%) cited that they did not receive the drugs. The primary reasons for not receiving them were taking medication for other ailments (19.3%), being exempted by the CDA due to old age (11.8%) and being out of station (6.4%). Additionally, 61 (32.6%) refused to accept the drugs due to various reasons. Of those offered with the drugs but did not consume them (n = 56), 45 (24.1%) reported fear of adverse effects as the reason for non-consumption. The same reason was attributed to 14.4% of 61 cases for the refusal to consume (Table 4).

**TABLE 4:** Reasons for non-participation in the last round of MDA (N = 187).

Non-participation in MDA	Reason	n (%)
Drugs not received/given by CDA (n = 70, 37.4%)	Out of station	12 (6.4)
	Taking drugs for other ailments	36 (19.3)
	Old age (exempted by CDA)	22 (11.8)
Refused to receive drugs (n = 61, 32.6%)	Not interested in taking drugs	16 (8.6)
	Fear of side effects	27 (14.4)
	On treatment for other illness	18 (9.6)
Drug received but not consumed (n = 56, 30%)	Fear of adverse events	45 (24.1)
	On treatment of other illness	9 (4.8)
	Not interested in taking drugs	2 (1)
Total (drug not consumed)		187

### Systematic non-participation

In this study, 52 (16.5%) individuals were considered as systematic non-compliers as they never participated in any of the MDA rounds. The most common reason given by systematic non-compliers was the fear of side effects (42.3%), followed by taking drugs for other ailments (23.0%). However, a small proportion (15.4%) of them said that they did not receive the drugs from the CDA. The other reasons for not participating in the MDA programme include not being interested (13.5%) and being out of station during the drug distribution (5.8%).

### Associated factors with non-participation in MDA

The associated factors with non-participation in MDA, was analysed using logistic regression models (Table 5). On univariate analysis, area of residence, knowledge on LF, status of other chronic diseases, uncertainty on the safety of drugs and neighbours’ participation were significantly associated with non-consumption among the participants. Individuals from rural areas showed higher consumption rates compared to peri-urban counterparts (OR = 2.68; *p* = 0.001). Individuals with chronic diseases such as diabetes (OR = 2.41; *p* = 0.010) and hypertension (OR = 1.77; *p* = 0.03) avoided MDA drugs. A higher knowledge score (more than 3) level was positively associated with participation (OR = 2.43, p-value <0.001). Faith on drug safety strongly influenced consumption (OR = 14.82; *p* < 0.001). Consumption by other family members (OR = 2.36, p-value = 0.019) and neighbours (OR = 4.84, p-value <0.001) influenced others to take the drug without hesitation. Other factors such as gender, religion, and education level did not show any significant association with drug consumption.

**TABLE 5:** Factors associated with drug consumption (univariate logistic regression).

Variable	Consumption		*Unadjusted OR (95% CI)*	*p-value*
	*Yes*	*No*		
*Gender* Female Male	89 (42.4) 39 (37.1)	121 (57.6) 66 (62.9)	1.00 1.24 (0.77–2.01)	− 0.373
*Residence* Rural Peri-urban	110 (45.8) 18 (24.0)	130 (54.2) 57 (76.0)	1.00 2.68 (1.49–4.82)	− 0.001
*Religion* Hindu Non-Hindu	111 (42.9) 17 (30.4)	148 (57.1) 39 (69.6)	1.00 1.72 (0.92–3.20)	− 0.087
*Education* Literate Illiterate	22 (46.8) 106 (39.6)	25 (53.2) 162 (60.5)	1.00 1.34 (0.72–2.51)	− 0.351
*Diabetes* No Yes	115 (43.9) 13 (24.5)	147 (56.1) 40 (75.5)	1.00 2.41 (1.23–4.71)	− 0.010
*Hypertension* No Yes	100 (44.4) 28 (31.1)	125 (55.6) 62 (68.9)	1.00 1.77 (1.05–2.97)	− 0.030
*Knowledge score* >3 ≤3	91 (49.2) 37 (28.5)	94 (50.8) 93 (71.5)	1.00 2.43 (1.51–3.92)	− <0.001
*Perceived seriousness of the disease* Yes No/Unsure	110 (43.3) 18 (29.5)	144 (56.7) 43 (70.5)	1.00 1.82 (1.00–3.34)	− −0.051
*Perceived risk of disease* Yes No/Unsure	44 (54.3) 84 (35.9)	37 (45.7) 150 (64.1)	1.00 2.12 (1.27–3.54)	− 0.004
*Perceived safety of the drugs* Yes No/Unsure	99 (73.9) 29 (16.0)	35 (26.1) 152 (84.0)	1.00 14.82 (8.52–25.78)	− <0.001
*Consumption among the family members* Yes No/Unsure	117 (43.3) 11 (24.4)	153 (56.7) 34 (75.6)	1.00 2.36 (1.15–4.86)	− 0.019
*Consumption among neighbours* Yes No/Unsure	103 (54.5) 25 (19.8)	86 (45.5) 101 (80.2)	1.00 4.84 (2.87–8.16)	− <0.001

Multivariate analysis showed that individuals residing in peri-urban areas (OR = 6.80, *p* < 0.001), those with chronic diseases such as diabetes (OR = 2.89, *p* = 0.018), individuals who perceived the drugs as unsafe (OR = 16.27, *p* < 0.001), and those whose neighbours did not consume the drugs (OR = 5.35, *p* < 0.001) were more likely to be non-compliant (Table 6).

**TABLE 6:** Factors associated drug consumption (multivariate logistic regression).

Variable	Compliance		Total (n = 315)	Adjusted	
	*Yes (n = 128)*	*No (n = 187)*		*OR (95% CI)*	*p-value*
*Residence* Rural Peri-urban	110 (45.8) 18 (24.0)	130 (54.2) 57 (76.0)	240 75	1.00 6.80 (3.02–15.31)	− <0.001
*Diabetes* No Yes	115 (43.9) 13 (24.5)	147 (56.1) 40 (75.5)	262 53	1.00 2.89 (1.20–6.95)	− 0.018
*Perceived safety of the drugs* Yes No/Unsure	99 (73.9) 29 (16.0)	35 (26.1) 152 (84.0)	134 181	1.00 16.27 (8.50–31.12)	− <0.001
*Consumption among neighbours* Yes No/Unsure	103 (54.5) 25 (19.8)	86 (45.5) 101 (80.2)	189 126	1.00 5.35 (2.69–10.63)	− <0.001

## Observation with drug administrators

The following is the summary of qualitative observations with CDAs in treating the elderly population:

• Lack of familial support system for the elderly: In certain areas, seasonal migration of younger family members to major cities like Mumbai and Bengaluru for employment, left the elderly population to make their own decision to participate in MDA. Therefore, the absence of reliable support led to anxiety among the elderly, deterring their participation in MDA due to fears of adverse reactions without immediate access to assistance.
• Higher rates of drug refusal: Some of the CDAs reported that elderly individuals often refuse the drugs due to their perception that LF is not a serious disease.
• Fear of health complications due to co-morbidities: Systemic illnesses such as hypertension, diabetes, cardiovascular diseases, etc. are common among the elderly population in the study area and they are on regular medications for other diseases. Therefore, drug administrators often hesitate to administer the MDA drugs to them due to concerns about potential drug interactions.
• Shortage of CDAs and higher coverage targets: Due to shortages and higher targets in resource-constrained areas, reduces the ability to effectively administer the MDA.
• Influence of political decisions: In some villages, the local community leaders directed the CDAs not to provide the drugs to the elderly population due to the fear of enhanced adverse effects, thereby limiting the treatment.
• Lack of awareness and risk perception about the disease: The CDAs feel that the risk perception of LF-disease is low among the elderly population in most areas.
• Absence of clear treatment guidelines: Absence of guidelines to treat the elderly population makes the CDAs hesitant to make treatment decisions.
• Inadequate community preparations: CDAs feel that prior educational and awareness campaigns are not adequate for community preparation.

## Discussion

The present study delves into the crucial yet understudied aspect of elderly participation in LF-MDA campaigns and investigates the underlying reasons for non-participation. The study offered an opportunity to assess knowledge and perception of the elderly regarding LF and MDA. Despite a significant proportion of the participants being familiar with LF, more than half (54%) lacked knowledge about the disease and preventive measures. Previous studies suggest that perceptions of LF and MDA can vary significantly between age groups, with differing impacts on drug consumption (Krentel and Wellings, 2018; Niles et al., 2021). Generally, advanced knowledge and positive perception of LF and MDA are associated with higher consumption rates (Ramaiah et al., 2006; Cantey et al., 2010; Hussain et al., 2014). The knowledge gap is particularly concerning given the study district, Yadgir was under MDA for over 16 years (Tripathi et al., 2022). The ongoing challenges in awareness highlights the necessity of more effective socio-behavioural change communication (SBCC) efforts in the region to enhance awareness of the disease, drug compliance, and undertaking preventive measures against mosquito vectors (Banerjee et al., 2019; Krentel et al., 2013). Building upon previous studies highlighting low health literacy among the elderly in India (Sahoo et al., 2021), our finding underscores the importance of tailored educational interventions to bridge this gap. Furthermore, while a significant proportion of the participants recognised the severity of LF, a striking majority (74.9%) did not perceive themselves at risk of infection. This dogma could impede drug compliance among them, emphasizing the need for personalized risk communication strategies for the elderly.

In this study, the never-treated elderly population was about 16.5%, leaving 83% of the people who had participated in at least one round of MDA. However, the coverage in the last round of MDA in 2023 was only 58.4%, with only 41% of respondents consumed the drug, falling below the target coverage (=consumption) of 65%. A similar observation was reported from other studies that poor MDA compliance among the elderly was due to systematic non-compliance (Nandha et al., 2007; Smaje et al., 2018). However, this report is in contrasts with many other studies showing that older adults tend to have higher compliance with MDA due to increased health awareness and greater exposure to health programmes (Kim et al., 2019; Njomo et al., 2020). The younger individuals often exhibit lower compliance rates, influenced by differing health beliefs (Boyd et al., 2010; Ranganath, 2010; Abd Elaziz et al., 2013; Chen et al., 2014).

Moreover, previous studies have reported that women are less likely to comply with MDA programs due to factors such as a lack of risk perception, increased adverse events, pregnancy, and family roles (Hussain et al., 2014; Abd Elaziz et al., 2013; Chen et al., 2014). This contrasts with our findings, which show that drug consumption among females was slightly higher (42.5%) compared to males.

The non-compliers of MDA remain untreated, and if infected, could serve as a reservoir of infection, contributing to transmission. Given these circumstances, achieving the elimination target poses a programmatic challenge (Coutts et al., 2017; Willis et al., 2020). As India has targeted the elimination of the disease by 2030, it becomes imperative to devise tailored strategies to track the never-treated or missed-out individuals in MDA and give special attention to convincing them on an individual basis.

Various reasons were cited for not participating in MDA, with concerns about the drug safety being a major factor. A significant proportion of participants (50.5%) were uncertain about the safety of MDA drugs, and 7% believed the drugs were unsafe. Trials of the triple-drug regimen (IDA) have generally found it safe for the elderly, with minimal side effects reported (Thomsen et al., 2016; King et al., 2018; Weil et al., 2019). A 2017 IDA trial in India showed that IDA is both effective and safe for mass treatment, with adverse effects being rare and typically mild (Jambulingam et al., 2021). Moreover, WHO guideline recommended treatment for all individuals in endemic areas, including the elderly, except for those who are severely ill (World Health Organization, 2019). However, concerns about drug interactions and age-related health risks persist, particularly in the elderly population. It is essential for healthcare providers to clearly communicate the safety profile of MDA and address specific concerns related to chronic conditions or other medications. Efforts are therefore necessary to instil trust in the quality, efficacy and safety of the drugs among the community. The involvement of medical officers is necessary in convincing the outliers to become compliers. In addition, experience shared by neighbour’s gives confidence to the people, and could improve drug compliance.

Overcoming the fear of adverse effects, reported by about 22.9% of respondents, is another potential challenge (Roy et al., 2013; Hussain et al., 2014). It is known that infection-specific adverse events are common in infected people when the parasites are cleared by medication (Budge et al., 2018). Strong messages are necessary during community mobilization to address this issue. Taking treatment for chronic diseases serve as another excuse for the elders to keep away from participating in the MDA (Abraham et al., 2024). Participants with systemic diseases such as diabetes and hypertension fear that the drugs may cause unintended health problems. Suitable measures need to be identified to handle this issue, ensuring that individuals with these conditions feel confident in participating in MDA without compromising their health.

Moreover, though supervised administration of drugs is recommended (World Health Organization, 2019), some proportions of the people are dispensed drugs for them to consume later and leave the drugs for the other absent family members. This practice may explain why a large number of the people did not consume the drugs left by the CDAs. We observed that drug administrators refrained from administering drugs to participants, with conditions such as diabetes, hypertension and other morbidities. Additionally, some older individuals were excluded from treatment due to concerns about age-related risks or potential drug interactions. This indicates that drug safety concerns are not only common among individuals but are also reinforced by drug distributors, potentially increasing fear within the community. Therefore, MDA treatment guidelines should address the concern of these left without treatment. Such guidelines will also strengthen the perception of the drug administrators and pave the way to treat the elderly.

## Top of form

Admittedly, this community-based study has a few limitations. The information provided is subjected to recall bias, as it relies on participant’s ability to accurately remember and report MDA participation. Cases reporting chronic morbidity could not be physically verified, which may introduce inaccuracies in the data regarding the health status of the participants. It was observed that women engaged in domestic chores were less attentive while answering the questions. These limitations should be taken into consideration when interpreting the findings of this study.

## Conclusion

The study identified a significant gap in the participation of the elderly population in LF-MDA programme even after several rounds of MDA. Factors associated with the consumption of drugs offer the need for a tailored approach to prepare the community, especially the elderly population, who are often ignored despite being at risk. Further qualitative socio-behavioural research is needed in different geographical settings to develop generic guidelines on the treatment of community, ensuring inclusivity and effectiveness across diverse populations. Such guidelines can enhance the engagement of all demographic groups, ultimately contributing to the success of LF elimination efforts.

## Data Availability

The raw data supporting the conclusions of this article will be made available by the authors, without undue reservation.
